# Importation of West Nile Virus Infection from Nicaragua to Spain

**DOI:** 10.3201/eid1407.071496

**Published:** 2008-07

**Authors:** Begoña Monge Maillo, Rogelio López-Vélez, Francesca Norman, Fernando de Ory, María Paz Sanchez-Seco, Cesare Giovanni Fedele

**Affiliations:** * Ramon y Cajal Hospital, Madrid, Spain; † Instituto de Salud Carlos III, Madrid

**To the Editor:** We report the case of a 51-year-old Spanish missionary who had lived Nicaragua (Managua) from 2004 to 2006. He had no other notable travel history during that period. In June 2006, he noticed malaise and nausea, followed by abrupt onset of fever (39°C), headache, cervical pain, and right hemiparesis. He was admitted to a local hospital in Nicaragua, at which time routine results of hematologic and biochemistry tests were within normal limits, except for mild neutrophilia. After cerebral magnetic resonance imaging (MRI), a diagnosis of ischemic cerebrovascular accident was made. He was treated with aspirin and ceftriaxone for an oropharyngeal infection.

Because neurologic symptoms persisted, 13 days later he was transferred to a hospital in Madrid, Spain. At that time, physical examination showed neck stiffness, a diminished level of consciousness, right flaccid hemiparesis, and facial weakness. Peripheral blood examination showed only mild neutrophilia. Cerebrospinal fluid (CSF) analysis showed a 65 mg/dL glucose level (blood glucose 140), proteins 136 g/dL, and 18 cells/mm^3^ (mainly lymphocytes). Serologic test results for HIV, hepatitis B virus, hepatitis C virus, syphilis, *Toxoplasma* spp*.,* and *Brucella* spp*.,* and CSF cultures for mycobacterial, bacterial, and fungal infections were all negative. Results of a computed tomographic scan of the brain were within normal limits. MRI showed nonspecific abnormal intensity of white matter signal. Electrophysiologic studies showed severe axonal motor neuropathy and moderate sensitive axonal neuropathy in the right upper limb. Gammaglobulin was administered intravenously for 5 days; the patient improved slightly. At discharge, the diagnosis was of “Guillain-Barré–like syndrome.” He was admitted to our Tropical Medicine Unit in Madrid, 160 days after onset of intial symptoms. West Nile virus (WNV) infection was suspected, and diagnostic tests were performed on all available samples. The first serum (S1) and CSF samples obtained 13 days after onset of symptoms were sent to us for testing. A second serum sample (S2) was obtained at 160 days.

The CSF was tested for flavivirus by using a generic PCR (*1*) and found negative, and for WNV immunoglobulin (Ig) G and IgM (Focus, Cypress, CA, USA) as previously described (*2*) and showed positive results for both immunoglobulins (Table). Serum samples were studied by ELISA for WNV IgG and IgM (Focus), and positive results were obtained for IgG to WNV in both samples and for IgM in S1. By a plaque reduction neutralization assay (PRNT) with 100 50% infection units of WNV (Eg-101 strain), positive titers of 256 in S1 and 64 in S2 were obtained (Table).

**Table:** Serologic results for antibodies to West Nile virus (WNV), dengue virus (DV), and tick-borne encephalitis virus (TBEV)*

Sample/ d after onset	WNV								DV ELISA			TBEV ELISA	
	ELISA					PRNT							
	IgG†	IgG‡	IT-IgG§	IgM†		Ab‡	IT Ab¶		IgG†	IgM†		IgG†	IgM†
S1/13	3.5	65,000	3.54	3.6		256	7.0		4.8	0.3		59.5	0.1
CSF/13		4,000		Pos#		32							
S2/160	3.2	16,000		0.8		64			4.5	0.2		27	0.1

*PRNT, plaque reduction neutralization assay; Ig, immunoglobulin; Ab, antibodies; IT, intrathecal; S1, first serum sample, obtained 13 days after onset of symptoms; CSF, cerebrospinal fluid; Pos, positive; S2, second serum sample, obtained 160 days after onset of symptoms. †Results expressed as the ratio of sample absorbance to cutoff value (positive >1.0). ‡Titer. §IT production of WNV IgG (ELISA). ¶IT production of WNV antibodies (PRNT). #CSF tested diluted 1:2.

S1 and CSF were subsequently titrated for WNV IgG by ELISA and for WNV antibodies by PRNT. Specific WNV IgG or total antibodies/albumin indices of 3.54 and 7.0, respectively, were obtained.

Serum samples were also assayed for IgG and IgM by ELISA against dengue virus (Panbio, Brisbane, Queensland, Australia) and tick-borne encephalitis virus (Siemens, Marsburg, Germany). Positive results were obtained for IgG to both viruses; titers did not vary, which suggests cross-reactivity with WNV or prior infection due to another flavivirus.

The causative role for WNV was confirmed by the following factors: 1) the detection of WNV-specific IgM, in the absence of IgM response to the other flavivirus, in combination with the variation of PRNT titer in S1 and S2; 2) the evidence of intrathecal WNV IgG by ELISA and WNV antibodies by PRNT, according to well established cutoff values (*3*); and 3) the detection of WNV-specific IgM in CSF. The final diagnosis was meningoencephalitis with acute flaccid paralysis due to WNV infection with right upper limb paraparesis and muscular atrophy as sequelae (Appendix Figure).

WNV is an arbovirus, family *Flaviviridae,* first detected in 1937. It is maintained worldwide in an enzootic cycle, transmitted primarily between avian hosts and mosquito vectors. Mosquitoes of the genus *Culex* are the main vectors. Humans and horses are accidental secondary hosts (*4*). WNV is now widely distributed in Africa, Asia, the Middle East, Europe, and the Americas. The first epidemics of WNV encephalitis were reported in the early 1950s in Egypt and Israel, then in France (1960s) and in South Africa (1970s). During the past 10 years, several WNV outbreaks in humans have been reported in the Mediterranean basin and in southern Europe (*5*,*6*). In the Americas, the first cases were reported in New York City in 1999 (*7*), and the spread of WNV to large areas of the United States, Canada, Mexico, Central America, and the Caribbean was demonstrated in subsequent years. The case reported here also represents a new case of imported WNV infection in Europe and documents an imported case acquired in Central America (*8*–*10*)

WNV infection in animals, mainly in birds and horses, has been documented in Mexico, the Caribbean, and areas of South America. Birds, in particular, have been implicated in spreading WNV during migratory events in Europe, Asia, Africa, and the Middle East. WNV could thus potentially be introduced by the same mechanism in Central and South America, resulting in possible transmission to humans in countries like Nicaragua (*7*).

In conclusion, the possibility should be considered of new cases of WNV infection arising outside classic areas of high risk. Clinicians should be aware of the possibility of imported WNV to request specific tests in symptomatic patients.

## Supplementary Material

Appendix Figure Right upper limb paraparesis and muscular atrophy as sequelae to West Nile virus infection in a 51-year-old man who had lived in Nicaragua.
