# Supplementary figures and images for: Importation of West Nile Virus Infection from Nicaragua to Spain
Source: Emerg Infect Dis. 2008 Jul;14(7):1171–3. doi: 10.3201/eid1407.071496 (PMC2600340; doi:10.3201/eid1407.071496)

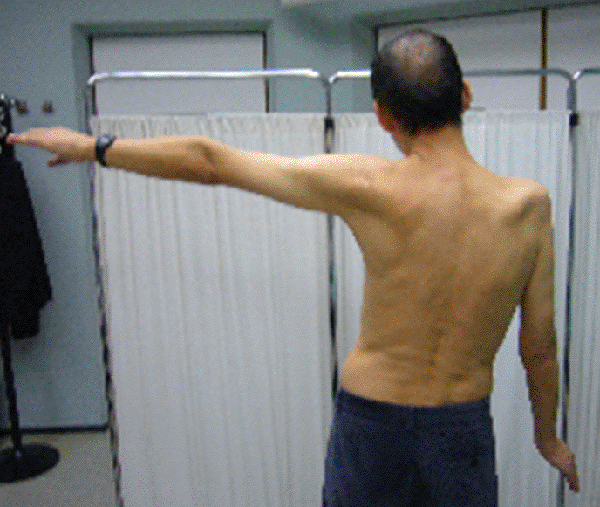

Supplement: Appendix Figure — Right upper limb paraparesis and muscular atrophy as sequelae to West Nile virus infection in a 51-year-old man who had lived in Nicaragua. [file 07-1496_app-s1.gif]
